# A public health approach to health workforce policy development in Europe

**DOI:** 10.1093/eurpub/ckaa123

**Published:** 2020-09-19

**Authors:** Natasha Azzopardi-Muscat

**Affiliations:** c1 Division of Country Health Policies and Systems, World Health Organization Regional Office for Europe, Copenhagen, Denmark; c2 Department of Health Services Management, Faculty of Health Sciences, University of Malta, Msida, Malta

Severe acute respiratory syndrome coronavirus 2 (SARS-CoV-2), better known as COVID-19, placed the health workforce at the centre of a dramatic series of events. As is so often the case in such circumstances, hype and sensationalism places a flitting spotlight on issues likely to make the media headlines, e.g. the tragic premature deaths of doctors and nurses caring for COVID-19 patients, the lack of personal protective equipment, or the strong support for ‘healthcare heroes’. Yet, the intensity and brevity of that focus is not well suited to tease out the complexity and chronicity of the challenges facing the health workforce in Europe today. COVID-19 exacerbated and unmasked gaps in health systems in Europe including:

- a cumulative shortage as a result of cuts to the health sector linked to the pursuit of blanket austerity policies implemented during preceding years;
- a lack of coordination between primary healthcare, hospitals and long-term care with unclear referral pathways;
- the primitive and patchy nature of digitalization penetration in health services resulting in digital quick-fix solutions to continue some semblance of care provision for those with chronic disease;
- depleted public health services unable to effectively surge capacity in a time manner;
- burnout amongst health workers;
- a huge dependence on health workers from outside the European region.

The physical and mental effects of COVID-19 on the health workforce at the level of individuals and organizations will become clearer as time goes by. The pressure on the health workforce comes from the burden of illnesses that stresses health system capacity as well as the ongoing risk of exposure and infection with COVID-19.^1^ For the time being, the pressure and burnout on healthcare professionals is likely to increase as health systems navigate delicately through the uncertainty of the COVID-19 pandemic transition phase. Health systems struggle to find the balance between remaining alert and able to deal with resurgence of the COVID-19 pandemic until such time that a vaccine may become available, whilst ensuring that routine services return to their full capacity. There is a pressing need to address pent-up demand as well as new demand both related to the sequelae of COVID-19 as well as the health impacts associated with the widespread economic downturn.^2^

Long before COVID-19 arrived to make Europe its epicentre propelling health workers into media headlines, the need to prioritize health workforce research and policy had been advocated by the health services epistemic community. Acknowledging that the polices and measures required to develop and sustain a fit-for-purpose health workforce in the 21st century European reality remain insufficiently understood, the European Public Health Association demonstrated foresight and leadership by setting up a section to focus on the health workforce in 2017 (https://eupha.org/health-workforce-research). Better integrating workforce education and training, implementing changes to skill mixes and competencies and monitoring and managing health workforce mobility were amongst the priorities identified.^3^ The need for investment in health workforce research as a means to ensure innovative models of services delivery across Europe was also highlighted as a priority in the Strategic Research Agenda for transforming innovation across health systems by the To-REACH project.^4^

Achieving universal health coverage, accelerating upward convergence in health outcomes and making substantive progress towards the sustainable development goals across the entire European Region necessitates a fresh, evidence-informed approach to health workforce policy (reference is here made to the European Region of the WHO which comprise 53 countries; https://www.euro.who.int/en/countries). The health labour market framework provides a useful starting point by identifying key influences on the dynamics of the health workforce, including the health needs of the population, the demand for health services and the supply and governance of health workers.^5^ Yet, these alone do not suffice. It is necessary to go beyond the numbers and to examine the changing nature of health services, the expectations of service users and the aspirations of the current and future health workforce. A public health approach to health workforce policy understanding the political dimension,^6^ combining ‘art and science’ and bringing together several disciplines may serve to deepen our understanding of the seemingly intractable shortages of health workers, which are currently experienced to varying extents by all countries.

The lessons learnt from COVID-19 presents a unique opportunity to reorient health workforce policy as an intricate component of future service planning and delivery, using a participatory approach to research and policy development with the voices of health workers and patients being as important as epidemiology and planning forecasts. There is an opportunity to understand whether health system efficiencies worth maintaining have been introduced. Digital uptake during this period has seen a marked increase. It is opportune to evaluate these experiences and understand to what extent they have been safe, effective and accepted by patients and professionals. Digital technologies are a promising solution to complement the scarcity of the health workforce so long as they are implemented within a strong governance framework with a key clear focus on keeping equity firmly in mind.^7^

Never has there been a better time to reinforce the quadruple aim for healthcare recognizing that health workforce engagement is a critical and necessary element for health systems transformation and furthermore, that such workforce engagement can only be achieved by improving the experience of providing care.^8^ This will clearly necessitate that mental health and psychosocial supports for the health workforce become a key priority for health systems in the coming years. Post-COVID-19 there is an opportunity for the world to recover, stronger and better. Similarly, there is an opportunity for us to develop health systems that are more resilient to external shocks and threats. Building resilient health systems can only be achieved by nurturing our health workforce. Equipping the health workforce with the knowledge, competencies and skills they need to deliver high quality health services is an essential requisite, but equally building trust and caring for our workforce as the human face of health systems is just as important.

## Disclaimer

The author is a staff member of the World Health Organization. The author alone is responsible for the views expressed in this article and they do not necessarily represent the decisions, policy or views of the World Health Organization.

*Conflicts of interest*: The author was the President of the European Public Health Association between November 2016 and March 2020.

## References

[1] Adams JG, Walls RM Supporting the health care workforce during the COVID-19 global epidemic. JAMA 2020;323:1439–40. 32163102 10.1001/jama.2020.3972

[2] WHO Regional Office for Europe. *Strengthening and Adjusting Public Health Measures Throughout the COVID-19 Transition Phases* Policy considerations for the WHO European Region, 24 April 2020 Available at: https://www.euro.who.int/en/health-topics/health-emergencies/coronavirus-covid-19/technical-guidance/2020/strengthening-and-adjusting-public-health-measures-throughout-the-covid-19-transition-phases.-policy-considerations-for-the-who-european-region,-24-april-2020 (14 June 2020, date last accessed).

[3] Kuhlmann E, Batenburg R, Wismar M, et al A call for action to establish a research agenda for building a future health workforce in Europe’. Health Res Policy Syst 2018;16:52. 29925432 10.1186/s12961-018-0333-x PMC6011393

[4] TO-REACH. Our strategic research agenda: TO-REACH transferring innovation in health systems. Available at: https://to-reach.eu/our-strategic-research-agenda (14 June 2020, date last accessed).

[5] Sousa A, Scheffler RM, Nyoni J, Boerma T A comprehensive health labour market framework for universal health coverage. Bull World Health Organ 2013;91:892–4. 24347720 10.2471/BLT.13.118927 PMC3853957

[6] Greer SL, Bekker MPM, Azzopardi-Muscat N, McKee M Political analysis in public health: middle range concepts to make sense of the politics of health. Eur J Public Health 2018;28:3–6. 10.1093/eurpub/cky194 PMC6209804 30383260

[7] Azzopardi-Muscat N, Ricciardi W, Odone A, et al Digitalization: potentials and pitfalls from a public health perspective. Eur J Public Health 2019;29:1–6. 10.1093/eurpub/ckz169 PMC6859515 31738438

[8] Sikka R, Morath JM, Leape L The quadruple aim: care, health, cost and meaning in work. BMJ Qual Saf 2015;24:608–10. 10.1136/bmjqs-2015-004160 26038586

